# A Case of Infantile Scurvy

**Published:** 1903-12

**Authors:** Bertram M. H. Rogers

**Affiliations:** Physician to the Royal Hospital for Sick Children and Women, Bristol.


### A CASE OF INFANTILE SCURVY.

BY

BERTRAM M. H. ROGERS, M.D., B.Ch., B.A. Oxon.

*Physician to the Royal Hospital for Sick Children and Women, Bristol.*

I SAW recently, in consultation with Mr. Morgan Thomas, an infant suffering from scurvy associated with rickets, and believe that the case is of sufficient rarity and interest to be recorded. It is the only case I have seen during my connection with the Children's Hospital, though I am told that one of my colleagues had a similar one under his care a few years ago. Further, I believe, they are not frequently seen at the larger institutions in this city.

The history is briefly as follows. The child was about nine months old, and had been since birth fed exclusively on sterilised milk and a proprietary food of wide renown. He had been out of sorts for a few days before I saw him, with indefinite symptoms, being fretful and crying readily. On the morning of the day Mr. Thomas asked me to see him, he had found the child very irritable and crying out if moved or handled at all, being evidently in great pain if touched. This was especially so when the legs were moved, the child preferring to lie with its legs hanging down in what is sometimes known as the paretic position. There was a large hemorrhage into one orbit, so large, that the eyeball was pushed downwards and forwards, rendering the child unable to close the eye. The gums were soft and had some hemorrhages on them, and the whole body was covered with the most severe and extensive sudamina I have ever seen, the chest and back being covered with clear blebs. The general condition of the child was fairly good; in fact, I have seen many children in the out-patient department with far more advanced rickets than this one. On examination I found the legs very tender to the touch, the child crying on the least movement. Both femora were enlarged with a fusiform swelling extending the whole length of the bone, but there was, as far as I could find, no hemorrhage between the shaft and the epiphyses, or crepitation that is sometimes found in these cases. There was also a similar swelling on one arm.

Considering the cause of the scurvy to be due to the diet, I suggested fresh milk should be substituted for sterilised, that the proprietary food be stopped altogether, and that the child should have orange juice daily. Mr. Thomas tells me that the child improved rapidly under this diet, though it had one or two relapses, and that it is now quite well.

The association of scurvy with rickets is usual but not necessary. Many cases have been reported in which rickets was not present, and even in children of more mature age scurvy has been seen from improper and insufficient food. As the cause of both is, as far as we know, due to an error in diet, the association of the two diseases is not surprising; in fact, considering the ignorance that exists among the poor—and the better-to-do, for that matter—about the proper bringing up of children, it is remarkable that scurvy is not more common, particularly since the extensive introduction of sterilised milk for quite young infants has become so prevalent.

In spite of what has been written on the subject, I am convinced that both boiling and sterilising alter the nutritive value of milk: its physical and chemical properties are changed, and it is for us to decide whether we shall run the risk of incurring a liability of contracting infectious diseases or possibly produce scurvy. My own inclinations lead me to take the latter risk, for after all scurvy seldom follows, and when it does occur is very amenable to treatment. So long as our sanitary authorities have no power of inspecting dairies outside their authority, or are unable to make some working arrangements with the county authorities, so long will there be a liability of infectious diseases being carried by milk.

The conclusions come to by the American Pediatric Society, from the study of 372 cases, of which 87 per cent. were in private, is (1) that the disease is produced by unsuitable food, and that a change of diet, even if it seems an improper one, may cure; (2) that proprietary foods are the chief cause; and (3) that the further such food is removed from the natural one for a child the more likely it is to be followed by the development of scurvy.

The experience of English physicians is the same, artificial foods and condensed milks being the most frequent causes.

Of the disease itself we know little beyond that it seems to be a pure blood disorder, and not depending on any disorder of the blood-forming organs, the spleen, or bone-marrow. The most recent theory appears to be that it is caused by ptomaines in food. It has been produced by feeding animals on decomposed food, and it is claimed that in recent Arctic expeditions it was this that caused it, and not the absence of fresh vegetables or fresh meat.

